# Sepsis in Internal Medicine: blood culture-based subtypes, hospital outcomes, and predictive biomarkers

**DOI:** 10.3389/fmed.2025.1503868

**Published:** 2025-01-30

**Authors:** Gaetano Zizzo, Gabriele Guazzardi, Daniela Bompane, Francesco Di Terlizzi, Giorgio Rotola, Ilario Stefani, Michela Medugno, Mario Bucalo, Antonino Mazzone

**Affiliations:** ^1^Department of Internal Medicine, Azienda Socio Sanitaria Territoriale (ASST) Ovest Milanese (Legnano—Cuggiono—Magenta—Abbiategrasso Hospitals), Milan, Italy; ^2^BIOMERIS (BIOMEdical Research Informatics Solutions), Pavia, Italy; ^3^Informative Systems, ASST Ovest Milanese, Milan, Italy

**Keywords:** Sepsis, COVID-19, internal medicine, culture-negative, polymicrobial, multidrug-resistant (MDR), outcomes, biomarkers

## Abstract

**Background:**

Sepsis is a challenging condition increasingly managed in medical wards, however literature and clinical evidence in this hospital setting are lacking.

**Methods:**

Using the computational i2b2 framework, we retrospectively analyzed data from patients admitted to internal medicine units of four hospitals in Lombardy (Italy) between January 2012 and December 2023, with a discharge diagnosis of sepsis, septic shock, or septicemia.

**Results:**

A total of 4,375 patients were recruited. Median length of stay (LOS) was 14 days, and mean ward-to-intensive care unit (ICU) transfer and in-hospital mortality rates were 11 and 26%, respectively; significant differences were observed over the years, with LOS peaks preceding mortality peaks by 1 year. Blood culture-negative sepses showed shorter stays and higher mortality (acute kidney injury and fast deterioration) compared to culture-positive ones; polymicrobial sepses showed higher ICU transfer rates (acute respiratory distress); while multidrug-resistant (MDR^+^) and/or polymicrobial sepses showed longer stays and higher mortality (complicated course) compared to drug-sensitive or monomicrobial ones. C-reactive protein elevation predicted rapidly evolving culture-negative sepsis, whereas lower leukocyte counts predicted prolonged hospitalization; higher fractions of inspired oxygen predicted polymicrobial sepsis, while lactate elevation predicted ICU transfer; ferritin elevation and increased leukocyte counts predicted MDR^+^ sepsis, while further ferritin elevation and decreased platelet counts predicted death. From 2016 to 2023, MDR^+^ sepsis frequency declined, due to decreased resistance to several antibiotic classes, such as cephalosporins, fluoroquinolones, and aminoglycosides; however, carbapenemase- and extended-spectrum beta-lactamase-producing Gram-negative bacteria, as well as vancomycin-resistant enterococci, increased, as did the frequency of polymicrobial sepsis following the COVID-19 outbreak.

**Conclusion:**

This work provides novel insights into sepsis management in internal medicine units, highlighting the need for validated biomarkers and implemented therapies in this scenario.

## Introduction

Sepsis is among the major global health concerns, in terms of morbidity, mortality and economic burden (1). It results from a dysregulated host response to infection, generating persistent, harmful inflammation with organ dysfunction and clinical deterioration, eventually progressing to refractory hypoperfusion, critical multiorgan failure, and death in a significant subset of patients (2).

The incidence of sepsis has increased in recent decades, in parallel with the increase in aging, chronic diseases and antimicrobial resistance (3–5). In fact, sepsis represents the main cause of in-hospital mortality, contributing directly or indirectly to at least half of all in-hospital deaths, as well as being responsible for long and complicated hospital stays, with frequent readmissions (5–7).

Sepsis is a highly heterogeneous and time-dependent life-threatening condition, thereby early identification of patients developing sepsis and at risk of progression is pivotal to optimize treatment strategies, prevent worse outcomes and reduce mortality (8). Most of the literature regarding the epidemiology, management, and prognosis of sepsis to date has focused on patients in emergency departments and intensive care units (ICU) (9, 10); however, owing to the aging of the general population and limited ICU resources, sepsis is increasingly managed in medical departments, where at least two-thirds of these patients are treated (7, 8, 11–16), but clinical evidence regarding the effectiveness of guidelines and bundles in this hospital setting is still largely lacking, and little is known about the characteristics and outcomes of patients with sepsis admitted to general medicine wards.

In this observational study, we investigated the clinical course of patients with sepsis admitted to four internal medicine units in Lombardy (Italy) over the last decade, focusing on key hospital clinical outcomes, such as length of stay (LOS), ward-to-ICU transfer and in-hospital mortality rates. Additionally, we stratified patients based on blood culture results, thereby looking for potential differences in clinical course between culture-positive versus culture-negative, polymicrobial versus monomicrobial, and multidrug-resistant (MDR) versus non-MDR sepsis patients, and we attempted to identify blood-based microbiological and laboratory biomarkers that could serve as potential predictors of distinct types of sepsis and outcomes. Finally, we studied the potential impact of the COVID-19 pandemic on sepsis and the trend of antimicrobial resistance in the latest years.

## Materials and methods

### Patients

We retrospectively analyzed patients hospitalized at Azienda Socio Sanitaria Territoriale (ASST) Ovest Milanese (i.e., Legnano, Cuggiono, Magenta, and Abbiategrasso hospitals, Milan, Italy) and admitted from the Emergency Department to the Internal Medicine wards between January 2012 and December 2023, with International Classification of Diseases, 9th revision-Clinical Modification (ICD9-CM) codes in their hospital discharge forms indicative of a diagnosis of “sepsis” (995.91 and 995.92), “septic shock” (785.52), or “septicemia” (038.0, 038.1, 038.3, 038.4, and 038.9) (17).

In general, codes relating to septicemia/bacteremia (038.-) or other primary infections detected by microbiological or instrumental tests (pneumonia, urinary tract infection, cholangitis, enterocolitis etc.) were entered as primary diagnosis, while complications of the primary infectious disease, such as sepsis or septic shock, were entered as secondary diagnosis. Sepsis was referred as an inappropriate and life-threatening host response to infection with hypotension and/or organ dysfunction (2) (a condition previously defined as “severe sepsis” and more correctly referred with code 995.92, even if code 995.91, referring to “sepsis” as infection-evoked systemic inflammatory response syndrome (18, 19), is still widely used). Septic shock was referred as a highly fatal condition of critical circulatory and cellular/metabolic abnormalities characterized by refractory hypotension requiring vasopressors or severe elevation in blood lactate level (2).

Clinical data [i.e., length of stay (LOS), intensive care unit (ICU) transfer, in-hospital mortality], microbiological data (i.e., positive or negative blood culture results, type and number of pathogens isolated, related antibiograms and number of antibiotic resistances), and additional laboratory data [i.e., leukocyte and platelet counts, fraction of inspired oxygen (FiO_2_) and arterial partial pressure of oxygen (PaO_2_)/FiO_2_ ratio, creatinine, alanine (ALT) and aspartate (AST) transaminases, bilirubin, C-reactive protein (CRP), ferritin, procalcitonin, and lactate levels at baseline] were collected.

Sepsis complicating an infectious disease but with no evidence of bloodstream infection (non-bacteremic sepsis) was defined as “culture-negative sepsis.” Culture-positive (bacteremic) sepsis with detection of two or more bloodstream pathogens was defined as “polymicrobial sepsis.” Culture-positive sepsis with detection of at least one bloodstream pathogen resistant to at least three antibiotic classes or more than six antibiotic drugs (considering on average one to three antimicrobials being tested for each class on the antibiograms) or to the majority of antibiotics (if fewer drugs were tested) was defined as “MDR^+^ sepsis.”

Data were obtained using the computational i2b2 (Informatics for Integrating Biology and the Bedside) framework (20) provided by BIOMERIS (BIOMEdical Research Informatics Solutions, Pavia, Italy).

The study conformed to the Declaration of Helsinki and was approved by the local Ethics Committee (Milano Area 3).

### Statistical analysis

For categorical (qualitative) variables, data were expressed as percentages; differences between two variables were analyzed using Fisher’s exact test, while multiple comparisons were analyzed using chi-square test. For continuous (quantitative) variables, data were expressed as mean (±standard deviation) or median; differences between two variables were analyzed using Mann–Whitney test, while multiple comparisons were analyzed using Kruskal–Wallis and Dunn’s *post-hoc* tests. Factors found to be significantly different in univariate analyses were included as potential predictors in multivariate analyses; best threshold values for logistic regression were defined using receiving operator characteristic (ROC) curves. *p*-values <0.05 were considered statistically significant. Data analysis and graphing were performed using Graphpad Prism^™^ 10 software (La Jolla, CA, United States).

## Results

### Length of stay, intensive care unit transfer and in-hospital mortality for sepsis

**Figure 1:**
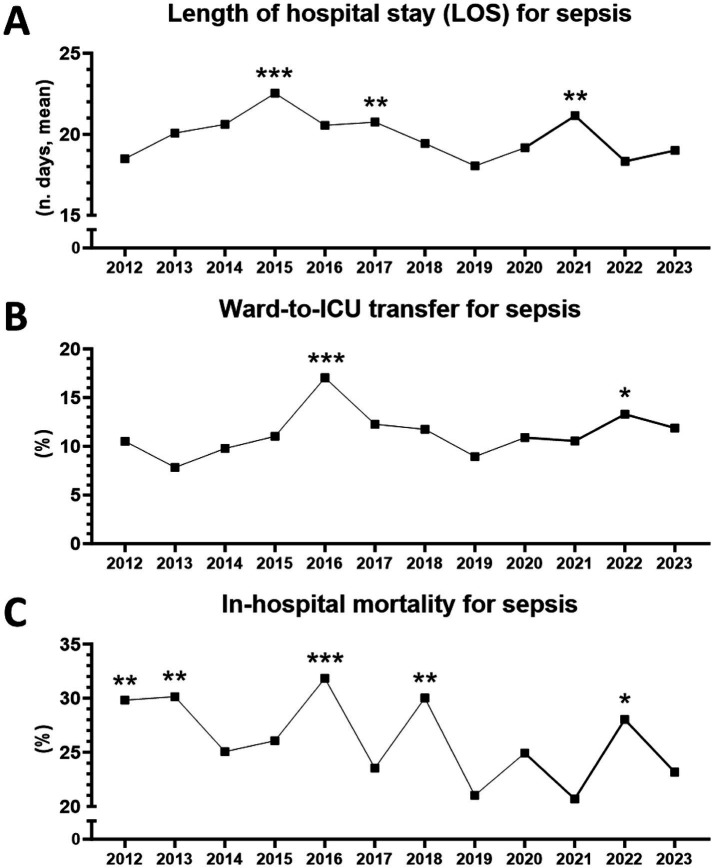
Hospital outcomes for sepsis in medical wards between 2012 and 2023 (*n* = 4,375 patients). **(A)** Length of stay (LOS). ^**^From 2012 to 2023, *p* = 0.0017 (Kruskal–Wallis test). ^***^2015 vs. 2012, *p* = 0.0001. ^*^2015 vs. 2022, *p* = 0.0449. ^**^2017 vs. 2012, *p* = 0.0044. ^**^2021 vs. 2012, *p* = 0.0021 (Dunn’s multiple comparisons). **(B)** Ward-to-ICU transfer. ^**^From 2012 to 2023, *p* = 0.0423 (chi-square). ^***^2016 vs. 2013, *p* = 0.0003. ^**^2016 vs. 2019, *p* = 0.0010. ^**^2016 vs. 2014, *p* = 0.0048. ^*^2016 vs. 2012, *p* = 0.0168. ^*^2016 vs. 2020, *p* = 0.0239. ^*^2016 vs. 2015, *p* = 0.0257. ^*^2016 vs. 2021, *p* = 0.0293. ^*^2016 vs. 2023, *p* = 0.0471. ^*^2016 vs. 2018, *p* = 0.0491. ^*^2022 vs. 2013, *p* = 0.0254 (Fisher’s exact test). **(C)** In-hospital mortality. ^**^From 2012 to 2023, *p* = 0.0031 (chi-square). ^***^2016 vs. 2019, *p* = 0.0009. ^**^2016 vs. 2021, *p* = 0.0031. ^**^2016 vs. 2023, *p* = 0.0071. ^*^2016 vs. 2017, *p* = 0.0249. ^**^2018 vs. 2019, *p* = 0.0030. ^*^2018 vs. 2021, *p* = 0.0101. ^*^2018 vs. 2023, *p* = 0.0252. ^**^2013 vs. 2019, *p* = 0.0038. ^*^2013 vs. 2021, *p* = 0.0111. ^*^2013 vs. 2023, *p* = 0.0257. ^**^2012 vs. 2019, *p* = 0.0050. ^*^2012 vs. 2021, *p* = 0.0116. ^*^2012 vs. 2023, *p* = 0.0323. ^*^2022 vs. 2019, *p* = 0.0239. ^*^2022 vs. 2021, *p* = 0.0453 (Fisher’s exact test).

A total of 4,375 sepsis patients were included in the analysis. Over the entire observation period, from 2012 to 2023, mean and median LOS for sepsis in medical wards was 19.7 (±20) and 14 days, respectively; statistical differences in LOS were observed over the years, with peaks occurring (in decreasing order) in 2015, 2021, and 2017 (Figure 1A).

Mean and median ward-to-ICU transfer for sepsis was 11.3% (±2.3) and 11%, respectively, with peaks observed in 2016 and 2022 (Figure 1B). Mean and median in-hospital mortality for sepsis was 26.2% (±3.7) and 25.6%, respectively, with peaks in 2016, 2018, 2013, 2012, and 2022. Overall, a trend towards lower mortality could be observed during the period (Figure 1C).

Of note, ICU transfer peaks coincided with mortality peaks, whereas LOS peaks alternated with them and seemed to anticipate mortality peaks by 1 year (Figures 1A–C).

### Impact of microbiological findings on sepsis severity and hospital outcomes

Microbiological data on blood cultures, available for 2,907 sepsis patients (since January 2016), allowed for patient stratification based on distinction between culture-positive vs. culture-negative, polymicrobial vs. monomicrobial, and MDR^+^ vs. non-MDR sepsis. In our series, we found that culture-negative patients had overall more severe sepsis than culture-positive ones, in terms of: higher inflammatory status, as assessed by increased leukocyte counts and CRP levels; higher levels of procalcitonin; greater tissue hypoperfusion and renal impairment, with higher lactate and creatinine levels; and more rapid and unfortunate evolution, with shorter hospital stays and higher mortality rates (Table 1 and Supplementary Figures S1A–F).

**Table 1:** Differences based on sepsis types (univariate analyses).

	Culture-positive	Culture-negative	*p*	Poly-microbial	Mono-microbial	*p*	MDR^+^	Non-MDR	*p*
Leukocyte count (10^3^/μL, median)	**9.4**	**9.7**	**0.0034**	9.5	9.3	0.6764	**10.2**	**8.7**	**0.0007**
CRP (mg/dL, median)	**10.0**	**11.9**	**<0.0001**	9.7	10.1	0.7094	**11.4**	**8.6**	**0.0016**
Ferritin (ng/mL, median)	455	449	0.7823	526	411	0.2878	**611**	**362**	**0.0196**
Procalcitonin (ng/mL, median)	**1.2**	**2.5**	**<0.0001**	1.4	1.2	0.2234	1.7	1.1	0.1337
Lactate (mmol/L, median)	**1.5**	**1.8**	**0.0365**	1.5	1.6	0.9386	1.6	1.5	0.6158
FiO_2_ (%, median)	**40**	**35**	**0.0039**	**50**	**40**	**0.008**	40	40	0.8472
PaO_2_/FiO_2_ ratio (mmHg, median)	200	226.7	0.9318	198.6	207.5	0.8946	182.0	193.8	0.4573
Creatinine (mg/dL, median)	**1.1**	**1.3**	**<0.0001**	1.2	1.1	0.7419	1.2	1.1	0.111
AST (U/L, median)	31	33	0.1791	30	32	0.8695	30	31	0.7572
ALT (U/L, median)	27	26	0.3049	27	27	0.8634	27	28	0.539
Bilirubin (mg/dL, median)	0.6	0.7	0.1774	0.7	0.5	0.136	0.6	0.6	0.5642
Platelet count (10^3^/μL, median)	179	178	0.4097	177	181	0.3466	181	178	0.2177
LOS (days, median)	**21**	**13**	**<0.0001**	**26**	**20**	**<0.0001**	**25**	**20**	**0.0007**
Ward-to-ICU transfer (%)	**16.3**	**10.5**	**<0.0001**	**26.7**	**12.5**	**<0.0001**	19.5	14.3	0.0745
In-hospital mortality (%)	**19.3**	**27.3**	**<0.0001**	**24.6**	**17.3**	**0.0316**	**24.0**	**16.5**	**0.0142**
MDR^+^ blood cultures (%)		—		**51.9**	**30.7**	**<0.0001**		—	
Polymicrobial blood cultures (%)		—			—		**37.0**	**19.5**	**<0.0001**

ALT, alanine transaminase; AST, aspartate transaminase; CRP, C-reactive protein; FiO_2_, fraction of inspired oxygen; ICU, intensive care unit; LOS, length of stay; MDR, multidrug-resistant; PaO_2_/FiO_2_, arterial partial pressure of oxygen (PaO_2_)/FiO_2_ ratio. Bold values are statistically significant.

On the other hand, culture-positive patients (*n* = 724, 24.9%), especially polymicrobial (*n* = 187, 25.8%) and MDR^+^ cases (*n* = 262, 36.2% of them), had a longer hospital stay. Whereas culture-negative sepsis was more frequently associated with acute kidney injury, culture-positive, particularly polymicrobial, sepsis was instead associated with acute respiratory distress syndrome (PaO_2_/FiO_2_ ratio ≤200 mmHg) and augmented oxygen and ventilation requirements, as assessed by higher FiO_2_ values and increased ICU transfer rates (Table 1 and Supplementary Figures S1G–L).

Among culture-positive patients, MDR^+^ cases had more severe inflammatory status, as assessed by higher circulating leukocyte, CRP and ferritin levels; and those with polymicrobial and/or MDR^+^ cultures had higher mortality rates compared to monomicrobial or non-MDR cases. Polymicrobial sepsis and MDR^+^ sepsis were found, in fact, to be closely associated conditions (Table 1 and Supplementary Figures S1M–R).

Patients with hospital stay shorter than 2 weeks or transferred to the ICU had higher in-hospital mortality. Patient groups with worse prognosis had increased procalcitonin, lactate, CRP and ferritin levels, higher leukocyte and lower platelet counts, altered indices of renal, respiratory and hepatic function, and more frequent detection of polymicrobial and/or MDR^+^ blood cultures (Table 2).

**Table 2:** Differences based on hospital outcomes (univariate analyses).

	LOS ≤14 days	LOS >14 days	*p*	ICU transfer	No transfer	*p*	Death	No death	*p*
Leukocyte count (10^3^/μL, median)	**10.0**	**9.2**	**<0.0001**	9.9	9.7	0.7392	**10.9**	**9.5**	**<0.0001**
CRP (mg/dL, median)	**12.6**	**9.8**	**<0.0001**	**14.2**	**11.11**	**0.0003**	**13.7**	**10.7**	**<0.0001**
Ferritin (ng/mL, median)	429	468	0.1763	**685**	**442**	**0.0008**	**701**	**440**	**<0.0001**
Procalcitonin (ng/mL, median)	**3.1**	**1.4**	**<0.0001**	**4.5**	**1.9**	**0.0014**	**4.0**	**1.8**	**<0.0001**
Lactate (mmol/L, median)	**1.8**	**1.5**	**0.0023**	**1.8**	**1.6**	**0.0224**	**2.2**	**1.5**	**<0.0001**
FiO_2_ (%, median)	**40**	**35**	**0.0262**	**40**	**26**	**<0.0001**	**40**	**35**	**0.0234**
PaO_2_/FiO_2_ ratio (mmHg, median)	210.8	222.5	0.4406	206.7	248.6	0.4794	190.4	232.5	0.0578
Creatinine (mg/dL, median)	**1.3**	**1.2**	**<0.0001**	1.3	1.2	0.5017	**1.7**	**1.1**	**<0.0001**
AST (U/L, median)	**36**	**31**	**0.0003**	**38**	**32**	**0.007**	**44**	**31**	**<0.0001**
ALT (U/L, median)	**27**	**26**	**0.0468**	**30**	**26**	**0.0104**	**32**	**26**	**0.0018**
Bilirubin (mg/dL, median)	**0.7**	**0.6**	**0.0338**	0.7	0.6	0.2428	**0.9**	**0.6**	**<0.0001**
Platelet count (10^3^/μL, median)	**171**	**186**	**<0.0001**	175	177	0.3735	**160**	**180**	**<0.0001**
LOS (days, median)		—		**21**	**14**	**<0.0001**	**7**	**16**	**<0.0001**
Ward-to-ICU transfer (%)	**7.7**	**14.9**	**<0.0001**		—		**18.1**	**8.8**	**<0.0001**
In-hospital mortality (%)	**35.4**	**16.5**	**<0.0001**	**42.2**	**24.1**	**<0.0001**		—	
MDR^+^ blood cultures (%)	30.8	38.4	0.0611	43.6	34.8	0.0745	**45.3**	**34.0**	**0.0142**
Polymicrobial blood cultures (%)	**19.4**	**28.5**	**0.0117**	**42.7**	**22.6**	**<0.0001**	**33.1**	**24.1**	**0.0316**

ALT, alanine transaminase; AST, aspartate transaminase; CRP, C-reactive protein; FiO_2_, fraction of inspired oxygen; ICU, intensive care unit; LOS, length of stay; MDR, multidrug-resistant; PaO_2_/FiO_2_, arterial partial pressure of oxygen (PaO_2_)/FiO_2_ ratio. Bold values are statistically significant.

### Predictors of sepsis types and outcomes

Potential predictive variables of distinct types of sepsis and hospital outcomes were sought and confirmed using multivariate analyses. Concerning sepsis types, it was found that: lower CRP levels and prolonged hospitalization were predictive of culture-positive sepsis; higher supplemental oxygen demand (FiO_2_) predicted culture-positive sepsis, particularly polymicrobial sepsis; elevated leukocyte counts and ferritin levels were predictive of MDR^+^ sepsis; and MDR^+^ cultures ultimately predicted polymicrobial sepsis (Table 3).

**Table 3:** Predictors of sepsis types (multivariate analyses).

Predicting factors	*β* coefficient	Standard error (SE)	Odds ratio (OR)	95% confidence interval (CI)	*p*-value
Culture-positive sepsis					
Leukocyte count >9,600/μL	0.2761	0.3488	1.318	0.6687 to 2.637	0.4286
**CRP >10.8 mg/dL**	−0.8513	0.3733	**0.4268**	0.2025 to 0.8805	**0.0226**
Procalcitonin >1.8 ng/mL	−0.3476	0.3674	0.7064	0.3432 to 1.458	0.3442
Lactate >1.7 mmol/L	−0.395	0.3565	0.6736	0.3322 to 1.351	0.2678
**Fi**O_2_ **>38%**	1.117	0.3664	**3.056**	1.513 to 6.399	**0.0023**
Creatinine >1.2 mg/dL	−0.03719	0.3652	0.9635	0.4736 to 1.993	0.9189
**LOS >17 days**	1.29	0.3952	**3.633**	1.715 to 8.143	**0.0011**
Ward-to-ICU transfer	0.4563	0.3526	1.578	0.7888 to 3.159	0.1957
Polymicrobial sepsis					
**Fi**O_2_ **>48%**	1.155	0.4727	**3.174**	1.277 to 8.245	**0.0146**
**MDR**^+^ **cultures**	1.085	0.4744	**2.96**	1.190 to 7.738	**0.0222**
LOS >23 days	0.1822	0.512	1.2	0.4435 to 3.366	0.7219
Ward-to-ICU transfer	0.2309	0.4783	1.26	0.4954 to 3.275	0.6292
MDR^+^ sepsis					
**Leukocyte count >9,500/μL**	0.8642	0.3635	**2.373**	1.172 to 4.900	**0.0174**
CRP >10.3 mg/dL	0.06388	0.3619	1.066	0.5202 to 2.161	0.8599
**Ferritin >456 ng/mL**	0.7574	0.3533	**2.133**	1.073 to 4.306	**0.0321**
Polymicrobial cultures	0.2489	0.3805	1.283	0.6072 to 2.716	0.513
LOS >22 days	0.624	0.4315	1.866	0.8062 to 4.423	0.1481
Ward-to-ICU transfer	0.7354	0.3892	2.086	0.9784 to 4.533	0.0588

CRP, C-reactive protein; FiO_2_, fraction of inspired oxygen; LOS, length of stay; ICU, intensive care unit; MDR, multidrug-resistant. For culture-positive sepsis: area under the ROC curve (AUC) 0.7406, 95% CI 0.6667 to 0.8144, *p* < 0.0001. For polymicrobial sepsis: AUC 0.6975, 95% CI 0.5900 to 0.8050, *p* = 0.0018. For MDR+ sepsis: AUC 0.6978, 95% CI 0.6166 to 0.7789, *p* < 0.0001.

In regard to major clinical outcomes, it was found that: polymicrobial sepsis and lower leukocyte counts were predictive of prolonged hospitalization (LOS >14 days); higher lactate levels predicted ICU transfer; while elevated ferritin levels, reduced platelet counts and faster evolution (LOS <14 days) were highly predictive of in-hospital mortality [area under the ROC curve (AUC) = 0.8659; 95% confidence interval (CI): 0.7747 to 0.9572; *p* < 0.0001] (Table 4).

**Table 4:** Predictors of hospital outcomes (multivariate analyses).

Predicting factors	*β* coefficient	Standard error (SE)	Odds ratio (OR)	95% confidence interval (CI)	*p*-value
LOS >14 days					
**Leukocyte count >9,700/μL**	−1.149	0.569	**0.3169**	0.09657 to 0.9259	**0.0434**
CRP >11.1 mg/dL	−0.274	0.546	0.7603	0.2501 to 2.186	0.6158
Procalcitonin >2 ng/mL	0.3751	0.5573	1.455	0.4884 to 4.428	0.5009
Lactate >1.7 mmol/L	−0.5741	0.5396	0.5632	0.1887 to 1.603	0.2874
Creatinine >1.2 mg/dL	−0.5501	0.5486	0.5769	0.1895 to 1.669	0.316
AST >34 U/L	0.4014	0.533	1.494	0.5294 to 4.370	0.4514
Platelet count >177,000/μL	0.1969	0.5258	1.218	0.4327 to 3.467	0.7081
**Polymicrobial cultures**	1.34	0.6791	**3.82**	1.140 to 17.71	**0.0484**
Ward-to-ICU transfer	0.3622	0.5543	1.436	0.4992 to 4.535	0.5135
Ward-to-ICU transfer					
Leukocyte count >9,700/μL	−0.249	0.5717	0.7796	0.2457 to 2.363	0.6632
CRP >12.5 mg/dL	−0.3157	0.5533	0.7293	0.2424 to 2.162	0.5683
Ferritin >570 ng/mL	−0.1008	0.5876	0.9041	0.2809 to 2.874	0.8638
Procalcitonin >3.1 ng/mL	0.5407	0.5673	1.717	0.5628 to 5.313	0.3406
**Lactate >1.8 mmol/L**	1.164	0.5862	**3.203**	1.040 to 10.60	**0.0471**
AST >36 U/L	−0.09911	0.558	0.9056	0.2994 to 2.723	0.859
Polymicrobial cultures	0.2385	0.5593	1.269	0.4227 to 3.864	0.6698
LOS >17 days	0.6115	0.9005	1.843	0.3230 to 12.14	0.4971
In-hospital mortality					
Leukocyte count >10,000/μL	0.6249	0.8742	1.868	0.3493 to 11.81	0.4747
CRP >11.9 mg/dL	−0.5811	0.8569	0.5593	0.09453 to 2.952	0.4977
**Ferritin >553 ng/mL**	2.91	1.02	**18.37**	3.095 to 188.4	**0.0043**
Procalcitonin >2.9 ng/mL	−0.6525	0.898	0.5207	0.07720 to 2.834	0.4675
Lactate >1.8 mmol/L	0.6479	0.7801	1.912	0.4126 to 9.346	0.4063
Creatinine >1.4 mg/dL	1.005	0.8018	2.732	0.5692 to 14.10	0.2101
AST >37 U/L	−1.168	0.7855	0.311	0.05856 to 1.346	0.1371
**Platelet count >172,000/μL**	−2.09	1.023	**0.1237**	0.01330 to 0.7996	**0.041**
Polymicrobial cultures	0.9573	0.7575	2.605	0.6081 to 12.50	0.2064
MDR^+^ cultures	−0.05128	0.7795	0.95	0.1948 to 4.450	0.9476
**LOS >13 days**	−4.165	1.795	**0.01553**	0.0002267 to 0.3911	**0.0204**
Ward-to-ICU transfer	0.471	0.7927	1.602	0.3424 to 8.201	0.5524

AST, aspartate transaminase; CRP, C-reactive protein; LOS, length of stay; ICU, intensive care unit; MDR, multidrug-resistant. For LOS >14 days: area under the ROC curve (AUC) 0.7319, 95% CI 0.6220 to 0.8418, *p* = 0.0006. For ICU transfer: AUC 0.6964, 95% CI 0.5741 to 0.8186, *p* = 0.0048. For in-hospital mortality: AUC 0.8659, 95% CI 0.7747 to 0.9572, *p* < 0.0001.

### Potential impact of COVID-19 on sepsis types and outcomes

**Figure 2:**
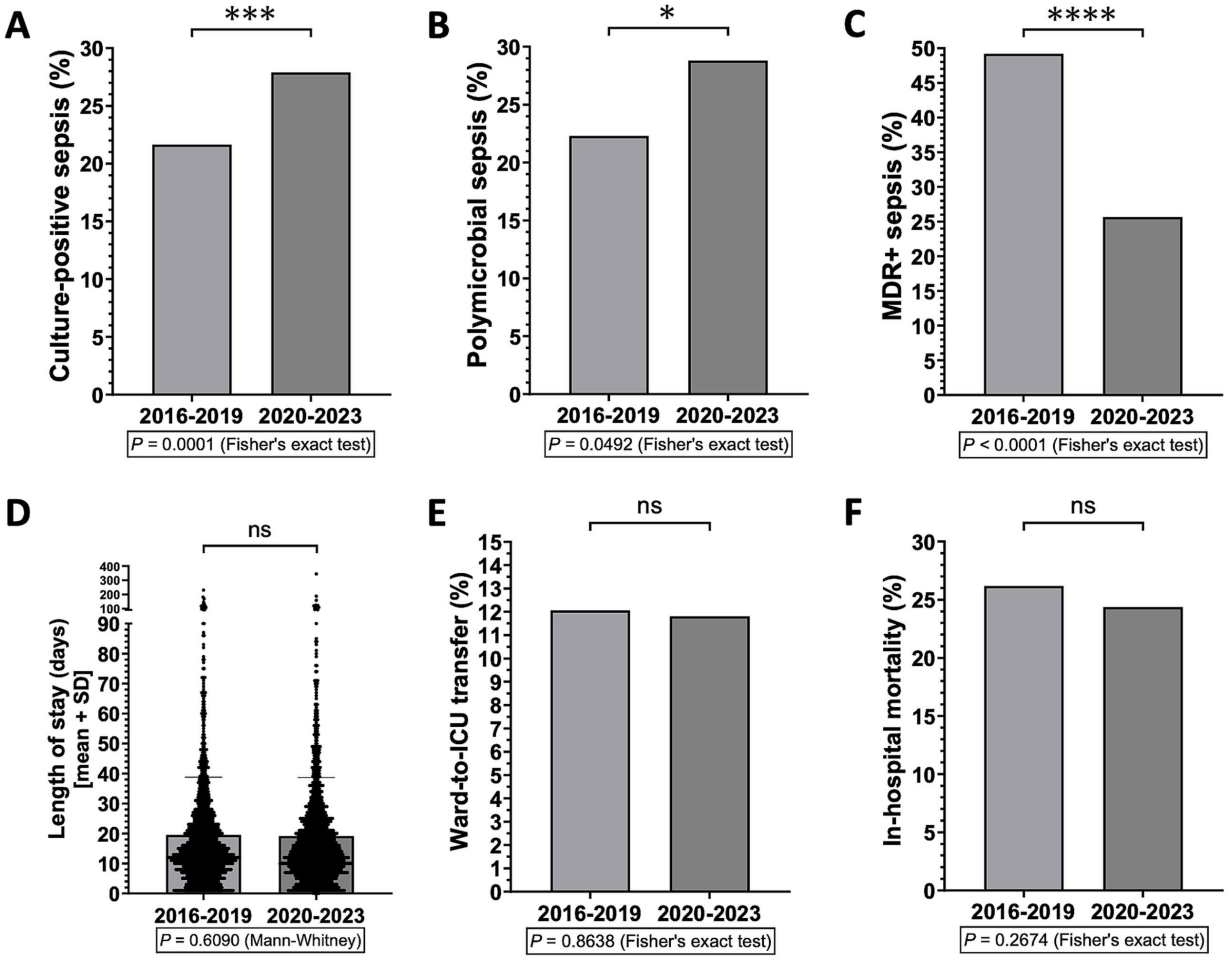
Differences in sepsis types and hospital outcomes for sepsis between before and after the outbreak of the COVID-19 pandemic (i.e., 2016–2019 vs. 2020–2023) (*n* = 2,907 patients). ^*^p < 0.05, ^***^p < 0.001, and ^****^p < 0.0001; ns, not significant.

Next, we investigated the possible effects of the recent COVID-19 pandemic on the frequency of different types of sepsis and on hospital outcomes for sepsis. To this end, we analyzed potential differences between the periods before and after the COVID-19 outbreak. We found that, in the COVID-19 era (i.e., 2020 to 2023), the frequency of culture-positive and polymicrobial sepsis has increased, yet the overall incidence of MDR^+^ sepsis has decreased, compared to the immediately preceding years (i.e., 2016 to 2019) (Figures 2A–C). In any case, no significant differences were observed in terms of LOS, ICU transfer or in-hospital mortality for sepsis (Figures 2D–F).

### Antibiotic resistance trends in Gram-negative and Gram-positive bacteria

**Figure 3:**
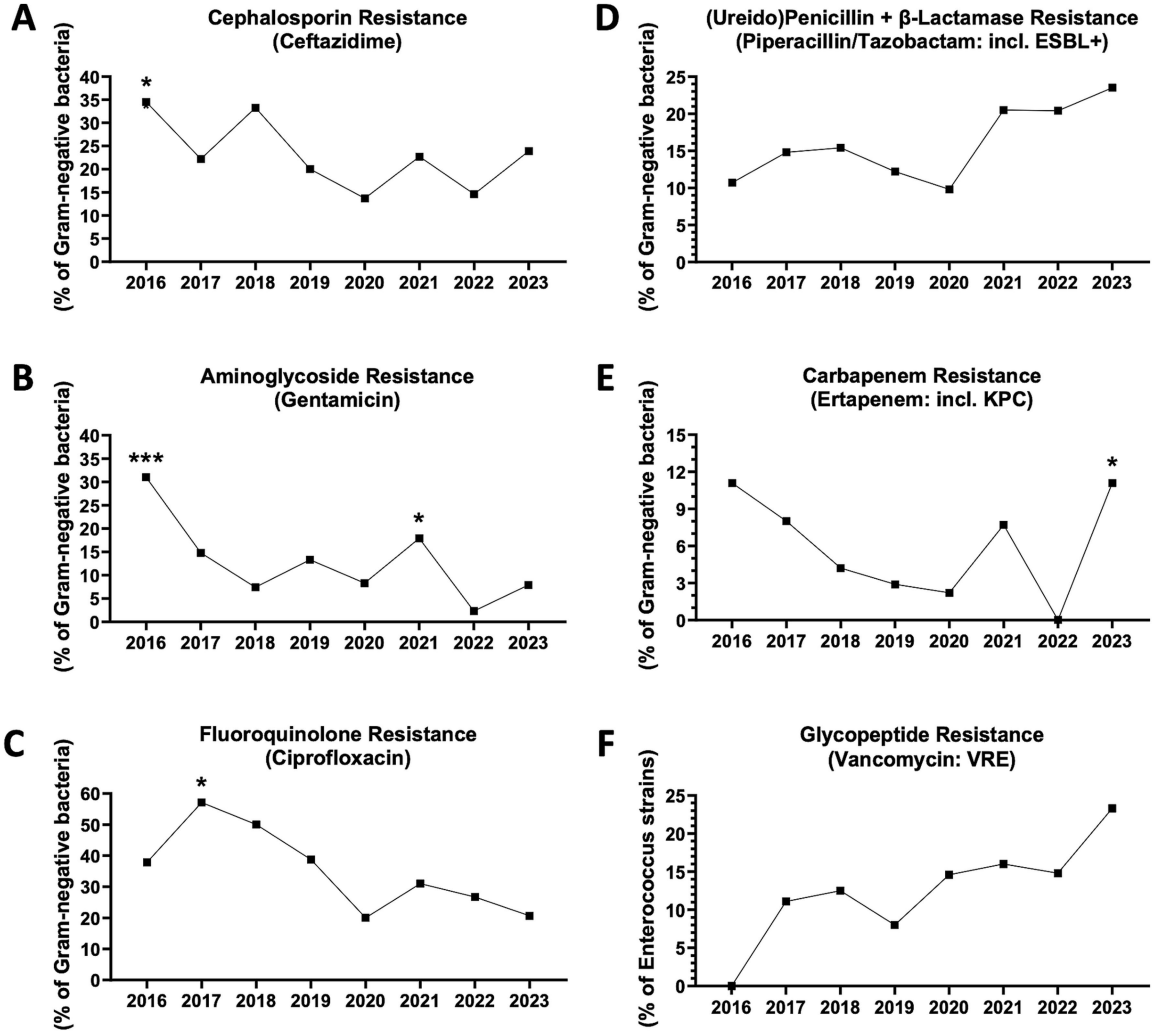
Antibiotic resistance trends in blood-isolated Gram-negative bacteria and enterococci. **(A)** Cephalosporin (ceftazidime) resistance (*n* = 368 antibiograms). ^*^2016 vs. 2020, *p* = 0.0453. 2016 vs. 2022, *p* = 0.0513. 2018 vs. 2020, *p* = 0.0742. 2018 vs. 2022, *p* = 0.0790 (Fisher’s exact test). **(B)** Fluoroquinolone (ciprofloxacin) resistance (*n* = 324). ^*^From 2016 to 2023, *p* = 0.0203 (chi-square). ^*^2017 vs. 2020, *p* = 0.0153. ^*^2017 vs. 2023, *p* = 0.0161. 2017 vs. 2022, *p* = 0.0528. ^***^2016–2019 vs. 2020–2023, *p* = 0.0007 (Fisher’s exact test). **(C)** Aminoglycoside (gentamicin) resistance (*n* = 351). ^*^From 2016 to 2023, *p* = 0.0140 (chi-square). ^***^2016 vs. 2022, *p* = 0.0008. ^*^2016 vs. 2020, *p* = 0.0138. ^*^2016 vs. 2018, *p* = 0.0420. ^*^2016 vs. 2019, *p* = 0.0485. ^*^2021 vs. 2022, *p* = 0.0244. 2019 vs. 2022, *p* = 0.0544. 2017 vs. 2022, *p* = 0.0690. ^*^2016–2019 vs. 2020–2023, *p* = 0.0484 (Fisher’s exact test). **(D)** Ureidopenicillin + β-lactamase (piperacillin/tazobactam) resistance (including extended spectrum β-lactamase producing bacteria or ESBL^+^) (*n* = 367). 2023 vs. 2020, *p* = 0.0568. 2023 vs. 2019, *p* = 0.0828 (Fisher’s exact test). **(E)** Carbapenem (ertapenem) resistance (including Klebsiella pneumoniae carbapenemase-producing bacteria or KPC) (*n* = 336). ^*^2023 vs. 2022, *p* = 0.0400. 2023 vs. 2019, *p* = 0.0842. 2016 vs. 2022, *p* = 0.0558 (Fisher’s exact test). **(F)** Glicopeptyde (vancomycin) resistant enterococci (VRE) (*n* = 177).

Finally, we studied the antimicrobial resistance of Gram-negative and Gram-positive germs *in vitro* by analyzing the antibiograms of pathogens isolated in the circulation of patients with culture-positive sepsis. In accordance with the overall trend of reduction in MDR^+^ sepsis reported above, a statistically significant reduction in resistance of Gram-negative bacterial strains to cephalosporins, quinolones and aminoglycosides was observed from 2016 to 2023 and in the period 2020–2023 compared to 2016–2019, with major peaks in 2016 or 2017 and nadirs in 2020 or 2022 (Figures 3A–C). However, in 2023, a new increase in the frequency of Gram-negative germs resistant to piperacillin/tazobactam (including extended-spectrum beta-lactamase-producing bacteria, ESBL^+^) or carbapenems (including *Klebsiella pneumoniae* carbapenemase-producing bacteria, KPC) was recorded, with a reversal of trend compared to 2020 or 2022, respectively (Figures 3D,E).

Regarding Gram-positive bacteria, the frequency of vancomycin-resistant enterococci (VRE) has progressively increased, although a statistical significance was not reached (Figure 3F). The frequencies of staphylococci and streptococci resistant to penicillins (including methicillin-resistant *Staphylococcus aureus*, MRSA), macrolides, quinolones and tetracyclines have tended to decline, until reaching a nadir in 2021 or 2022 (Supplementary Figures S2A–E). Nevertheless, in 2023, possible trend reversals were observed, with a particularly significant increase in tetracycline resistance (Supplementary Figure S2E).

## Discussion

This study provides novel insights into the epidemiological trends and clinical outcomes of sepsis in internal medicine units, an underexplored hospital setting, over a 12-year period. This work is also among the first to comprehensively analyze the prognostic significance of several laboratory variables in this environment, identifying key biomarkers, such as ferritin, that may predict adverse outcomes. These findings bridge a critical gap in sepsis management outside the ICU, where limited resources and an aging patient population represent unique challenges that necessitate improved diagnostic and therapeutic strategies.

Univariate analyses revealed discrete associations between sepsis types and hospital outcomes, in particular: culture-negative sepsis was associated with shorter stay and higher mortality (more pronounced hypoperfusion with acute kidney injury and rapid progression); polymicrobial sepsis was associated with higher ICU transfer rates (more pronounced hypoventilation with acute respiratory distress syndrome and higher need of oxygen and ventilation); while MDR^+^ and/or polymicrobial sepsis were associated with longer stay and higher mortality (complicated course).

Multivariate analyses confirmed reciprocal associations of culture-positive sepsis with prolonged hospitalization and oxygen requirement, specifically: MDR^+^ cultures predicted polymicrobial sepsis and the latter predicted longer hospital stay; in turn, a longer hospital stay predicted culture-positive sepsis, and higher oxygen need predicted culture-positive and polymicrobial sepsis. In accordance with our results, a recent meta-analysis showed that patients with culture-positive sepsis had longer duration of mechanical ventilation and longer hospital stay compared to culture-negative ones (21), while other authors reported that patients with polymicrobial sepsis had more need of ventilation and longer hospital stay compared to monomicrobial cases (15, 22).

Notably, we observed that hospitalizations with the highest LOS peaks precede those with the highest mortality peaks by 1 year. Hospitalizations and antibiotic therapies in previous months have been independently associated with occurrance of MDR^+^ septicemia at readmission (23, 24), and our data confirmed that polymicrobial sepsis often harbors MDR^+^ bacteria (e.g., VRE, carbapanem-resistant *Klebsiella* and other Gram-negative bacteria), which may ultimately be responsible for higher mortality (15, 25). In this regard, increased circulating levels of ferritin were herein found to predict both MDR^+^ sepsis and in-hospital mortality.

While on the one hand we have recorded an overall reduction in MDR^+^ sepsis (and in-hospital mortality) in recent years, due to a significant drop in the resistance of Gram-negative bacteria to cephalosporins, fluoroquinolones and aminoglycosides, with stability or at least numerical reduction of discrete resistant Gram-positive strains (e.g., MRSA), on the other hand we have highlighted that septicemia caused by carbapenemase-producing (e.g., KPC and others) and ESBL^+^ Gram-negative bacteria, as well as VRE, are on the rise, in agreement with other reports (24, 26, 27). The inappropriate use of broad-spectrum nosocomial antimicrobials, such as carbapenems, protected ureidopenicillins (e.g., piperacillin/tazobactam), and glycopeptides (e.g., vancomycin), to treat presumed bacterial coinfections during the COVID-19 pandemic (28) likely contributed to increase the resistance to these specific antibiotics. Moreover, the rise in polymicrobial sepsis herein observed in post-COVID years may reflect the immunosuppressive effects of SARS-CoV-2 among elderly patients (29). Nonetheless, the overall decrease in MDR^+^ rates seemed to result in a numerical reduction of sepsis mortality over the period 2016–2023, a trend that became fully significant when considering the entire observation period 2012–2023, in line with previous literature and meta-analyses (11, 30). In any case, there remains a need for further implementation of antibiotic stewardship programs in internal medicine units, with less protracted and more targeted therapies, in the aim to be more effective against sepsis but less harmful on antimicrobial resistance (7, 27, 31).

Another unmet need in the management of sepsis in medical wards is the lack of validated biomarkers of MDR^+^ sepsis and poor prognosis. Here, we identified circulating predictors of distinct types of sepsis and hospital outcomes, highighting differences between commonly used laboratory variables, such as leukocyte and platelet counts, CRP, procalcitonin, lactate, and ferritin levels. In particular, CRP elevation predicted rapidly evolving culture-negative sepsis, whereas decreased leukocyte counts predicted longer hospital stay; in accord with previous observations (32), lactate elevation predicted ICU transfer; and, most remarkably, ferritin elevation along with increased leukocyte counts were predictive of MDR^+^ sepsis, while further ferritin elevation along with decreased platelet counts were highly predictive of in-hospital mortality.

In our study, baseline procalcitonin was higher in culture-negative sepsis but, in agreement with other reports (8, 33), did not emerge as an independent predictor. Previous studies have highlighted that procalcitonin is useful outside the ICU for early recognition of sepsis, including culture-negative ones, from other inflammatory syndromes, while other indices (e.g., MR-proadrenomedullin) are instead more sensitive biomarkers of culture-positive forms; hence, baseline procalcitonin has an established diagnostic role in early sepsis (34, 35). Nevertheless, since procalcitonin level rapidly declines if antibiotic treatment is effective, its change within few days from admission (delta-procalcitonin) may have some prognostic role in sepsis patients admitted to internal medicine wards (33). In either case, serum procalcitonin levels are closely related to the presence or load of a bacterial pathogen. By contrast, serum ferritin levels are associated with the magnitude of the host inflammatory response, in particular macrophage activation (36, 37): in case the pathogen is resistant to treatment (e.g., first antibiotics administered in the emergency room), leukocytes such as macrophages and cytotoxic T cells will be aberrantly activated in the attempt to kill and clear it; if this response is protracted over time (e.g., in case of MDR^+^ pathogens), it may lead to macrophage activation syndrome-like features including hyperinflammation uncoupled to the pathogen load, macrophage dysregulation/reprogramming and hemophagocytosis, which ultimately account for thrombocytopenia and rapid progression to death (37–40). Recently, Fang et al. (41) observed that serum ferritin ≥591.5 ng/mL acts as an independent predictor of in-hospital mortality in ICU patients (OR 2.29, 95% CI 1.83 to 2.87, *p* < 0.001), with moderate predictive power (AUC = 0.651). Here we show that, in non-ICU patients, baseline ferritin >553 ng/mL is a much stronger independent predictor of in-hospital mortality (OR 18.37, 95% CI 3.095 to 188.4, *p* = 0.0043), with high predictive power (AUC = 0.866), thus suggesting its use as a key prognostic biomarker of sepsis in medical departments, even better than more traditional indices such as baseline lactate or procalcitonin.

The literature regarding culture-negative sepsis is still limited. Culture-negative sepsis is probably an underestimated entity, since patients without an identified bloodstream pathogen are less likely to obtain a sepsis diagnosis (42). Indeed, blood culture sampling has been reported to identify the causative organism of sepsis only in 15–30% of the patients (42, 43) and, accordingly, proportion of patients diagnosed with culture-positive sepsis in our series was only 24.9%. Culture-negative sepsis would represent at least half of total cases and its proportion is on the rise (16, 42, 44) but, despite its great prevalence, it is largely understudied and prognosis is somewhat controversial (42, 45). Our observation of higher in-hospital mortality in culture-negative sepsis aligns with previous reports by Gupta et al. (44), who noted that treatment remains empiric and often less effective in such cases. However, the debate continues as Mellhammar et al. (42) observed lower 90-day mortality in culture-negative cases, suggesting the need for further studies. On the other hand, it was recognized that culture-negative sepsis is more frequent in case of abdominal or pulmonary infections, rather than urinary ones (42), and the former actually show higher mortality than the latter (46, 47).

The rate of in-hospital mortality from sepsis in our experience was 26%, in line with global studies (3, 4, 48). Sepsis mortality changes depending on organ dysfunction: less than 20% in cases without organ dysfunction [“sepsis” according to Sepsis-1/2 criteria (18, 19)], between 20 and 50% in cases with organ dysfunction [“sepsis” according to Sepsis-3 criteria (2) or “severe sepsis” according to Sepsis-1/2 criteria], and over 50% in patients with septic shock (3). Fleischmann et al. (4) estimated that hospital mortality was 17% for “sepsis” and 26% for “severe sepsis” during the decade 2006–2015, whereas an updated and extended systematic review up to 2019 and meta-analysis of total patients with “sepsis,” defined according to clinical criteria (Sepsis-1/2/3) or relevant ICD codes, concluded that 26.7% of hospital-treated patients vs. 49.1% of ICU-treated patients died prior to discharge (48).

Our results also show several similarities with those reported in recent Italian studies focused on sepsis in medical wards (8, 16). In the SEMINA study, in particular, the composite outcome “in-hospital mortality or ICU transfer” was achieved in around 37% of sepsis patients (31.2% death + 5.3% ICU), as in our series (26% death + 11% ICU); furthermore, in accord with our observations, patients with culture-negative sepsis were the majority, with at least numerically higher mortality rates; and non-survivors had a shorter stay, most often less than 2 weeks, whereas a longer stay is highlighted here to significantly protect against in-hospital mortality (16). Finally, the subsets of patients who achieved the poorest outcomes had higher FiO_2_ requirements and lower PaO_2_/FiO_2_ ratios, higher creatinine levels, lower platelet counts, and higher frequencies of MDR^+^ cultures (8, 16).

Our work has several strengths. The first is certainly the large number of patients included in the study. The literature on the clinical course and prognosis of sepsis in medical wards is still scarse. Using the innovative i2b2 computational methodology, we were able to collect and analyze real-life data from up to 4,375 sepsis patients admitted specifically to internal medicine units. Second, we stratified patients based on blood culture findings, thus identifying interesting associations between microbiological and clinical characteristics, as well as useful circulating biomarkers predicting sepsis types and outcomes. Third, we studied the most recent epidemiological trends over broad time frames, examining key hospital outcomes, antimicrobial resistance and the potential impact of the COVID-19 pandemic on sepsis presentation and severity.

This study also has some limitations, including retrospective design and inclusion of patients according to ICD codes rather than Sepsis-3 or other clinical criteria (8, 16). Future studies should adopt standardised diagnostic criteria to ensure consistency. On the other hand, most of the studies conducted so far on sepsis in non-ICU settings were retrospective and based on ICD-coded hospital discharge databases, and no significant differences in mortality were found between sepsis patients identified based on ICD-codes vs. clinical criteria, nor based on Sepsis-1 vs. Sepsis-3 criteria (48). According to the third international consensus on sepsis and septic shock (Sepsis-3, 2006) (2), patients with a quick Sequential Organ Failure Assessment (qSOFA) ≥2 should be surveilled as at higher risk of in-hospital death. However, the most recent international guidelines (Survival Sepsis Campaign, 2021) (9) recommended against using qSOFA as a single screening tool for sepsis or septic shock compared to SIRS, National Early Warning Score (NEWS) or Modified Early Warning Score (MEWS), due to higher specificity but lower sensitivity in early identification of sepsis patients and prediction of sepsis outcomes, while the diagnosis of sepsis remains confirmed in case of a SOFA score ≥2. Although our analysis did not include clinimetric assessment of mental status, respiratory rate, heart rate, blood pressure, urine output and use of vasopressors for deriving SIRS, qSOFA or septic shock criteria (8, 14–16, 49), we did include all laboratory variables considered in the SOFA score. Other limitations of this study are lack of specific data about hospital-acquired sepsis (16, 50) and lack of statistical adjustments for confounding variables such as age, sex, comorbidities, sites of primary infection, number of blood culture sets, and timing of antibiotic therapy (42, 46, 47). In fact, comorbidities might importantly affect hospital outcomes as well as circulating levels of biomarkers.

In conclusion, despite some limitations, our study sheds new light on the clinical, epidemiological, microbiological and prognostic features of sepsis patients managed in internal medicine units. High-risk conditions such as culture-negative sepsis, polymicrobial sepsis and septicemia by ESBL^+^, carbapenemase-producers and VRE are on the rise, highlighting the need for new management strategies. Results suggest incorporating ferritin as a routine biomarker in risk stratification for sepsis patients in medical wards, where targeted interventions could be developed for high-ferritin patients to ameliorate their outcomes. Further prospective, multicenter studies are needed to validate the role of ferritin and other biomarkers in predicting outcomes and guiding treatment.

## Data Availability

The original contributions presented in the study are included in the article/Supplementary material, further inquiries can be directed to the corresponding author.

## Supplementary material

SUPPLEMENTARY FIGURE S1 Differences in clinical outcomes and laboratory indices based on blood culture results. **(A–F,J–K)** Culture-positive vs. culture-negative sepsis (*n* = 2,907 patients). **(G–I,L)** Polymicrobial vs. unimicrobial sepsis (*n* = 724). **(M–R)** Multidrug-resistant (MDR^+^) vs. non-MDR sepsis (*n* = 724). ^*^*p* < 0.05, ^**^*p* < 0.01, ^***^*p* < 0.001, and ^****^*p* < 0.0001; ns, not significant.

SUPPLEMENTARY FIGURE S2 Antibiotic resistance trends in other blood-isolated Gram-positive bacteria. **(A)** Penicillin (oxacillin or penillin G) resistant *S. aureus* (methicillin-resistant *Staphylococcus aureus* or MRSA) (*n* = 80 antibiograms). 2019 vs. 2021, *p* = 0.0785 (Fisher’s exact test). **(B)** Macrolide (erythromycin) resistant *S. aureus* (*n* = 77). **(C)** Fluoroquinolone (levofloxacin) resistant *S. aureus* (*n* = 78). **(D)** Macrolide (erythromycin) resistant staphilococci and streptococci (*n* = 331). **(E)** Tetracycline resistant staphilococci and streptococci (*n* = 351). ^**^From 2016 to 2023, *p* = 0.0015 (chi-square). ^****^2023 vs. 2021, *p* < 0.0001. ^**^2023 vs. 2020, *p* = 0.0034. ^*^2023 vs. 2022, *p* = 0.0324. ^***^2018 vs. 2021, *p* = 0.0009. ^**^2016 vs. 2021, *p* = 0.0010. ^**^2019 vs. 2021, *p* = 0.0012. ^**^2017 vs. 2021, *p* = 0.0015. ^*^2022 vs. 2021, *p* = 0.0117. ^*^2020 vs. 2021, *p* = 0.0282. 2016–2019 vs. 2020–2023, *p* = 0.0963 (Fisher’s exact test).
