# Supplementary figures and images for: Sepsis in Internal Medicine: blood culture-based subtypes, hospital outcomes, and predictive biomarkers
Source: Front Med (Lausanne). 2025 Jan 30;12:1503868. doi: 10.3389/fmed.2025.1503868 (PMC11822444; doi:10.3389/fmed.2025.1503868)

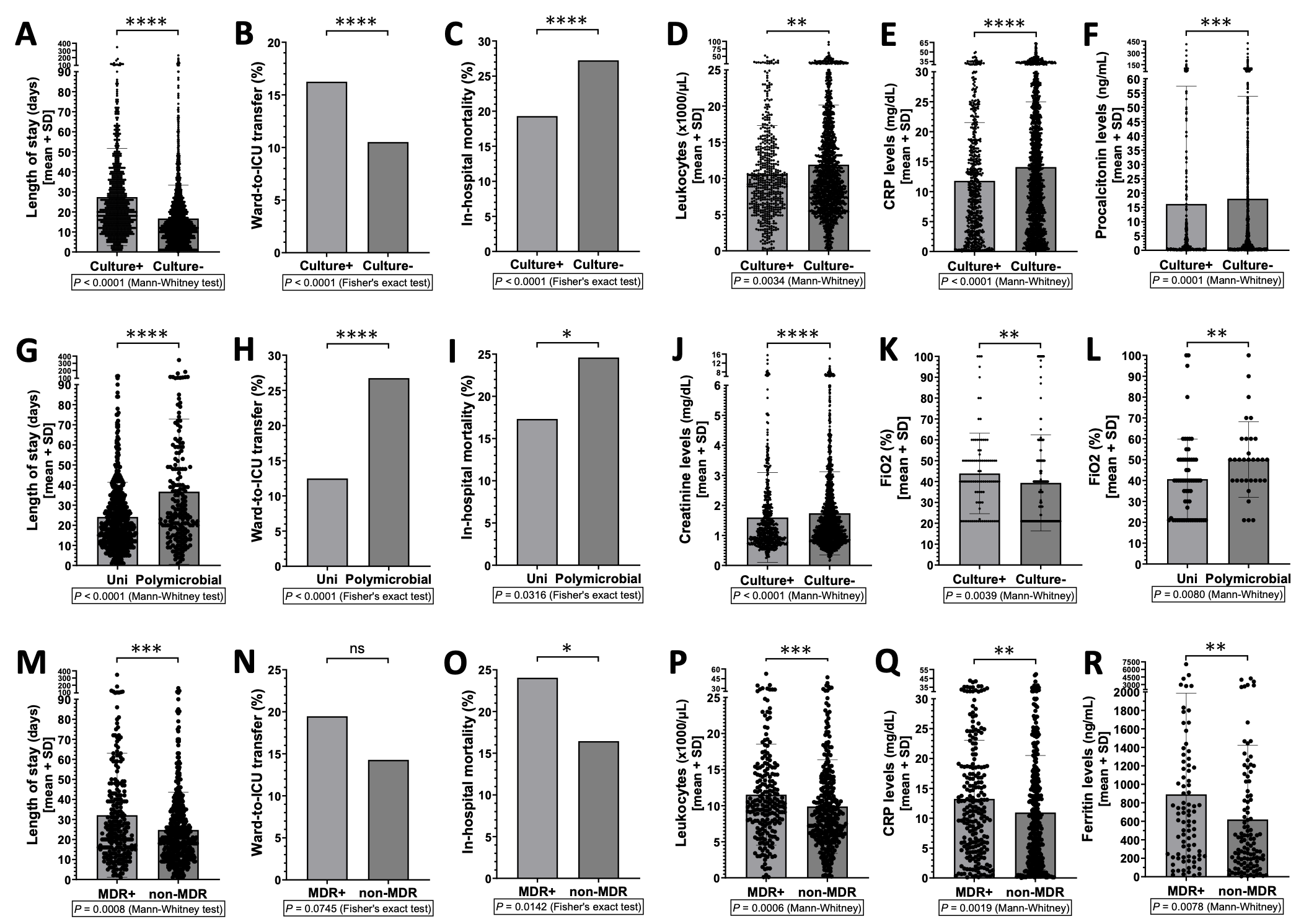

Supplement: SUPPLEMENTARY FIGURE S1 — Differences in clinical outcomes and laboratory indices based on blood culture results. (A–F,J–K) Culture-positive vs. culture-negative sepsis (n = 2,907 patients). (G–I,L) Polymicrobial vs. unimicrobial sepsis (n = 724). (M–R) Multidrug-resistant (MDR+) vs. non-MDR sepsis (n = 724). *p < 0.05, **p < 0.01, ***p < 0.001, and ****p < 0.0001; ns, not significant. [file Image_1.TIFF]

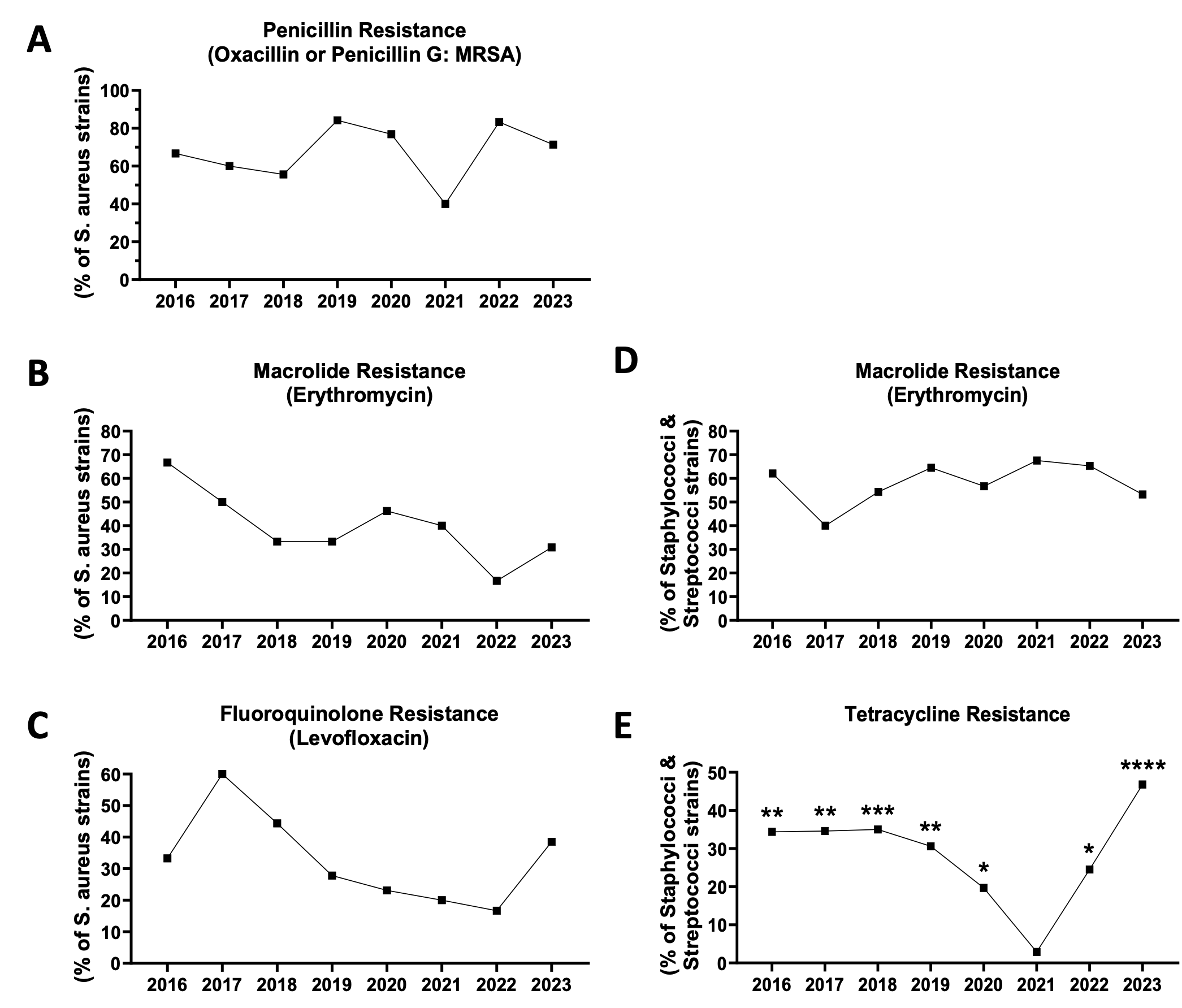

Supplement: SUPPLEMENTARY FIGURE S2 — Antibiotic resistance trends in other blood-isolated Gram-positive bacteria. (A) Penicillin (oxacillin or penillin G) resistant S. aureus (methicillin-resistant Staphylococcus aureus or MRSA) (n = 80 antibiograms). 2019 vs. 2021, p = 0.0785 (Fisher’s exact test). (B) Macrolide (erythromycin) resistant S. aureus (n = 77). (C) Fluoroquinolone (levofloxacin) resistant S. aureus (n = 78). (D) Macrolide (erythromycin) resistant staphilococci and streptococci (n = 331). (E) Tetracycline resistant staphilococci and streptococci (n = 351). **From 2016 to 2023, p = 0.0015 (chi-square). ****2023 vs. 2021, p < 0.0001. **2023 vs. 2020, p = 0.0034. *2023 vs. 2022, p = 0.0324. ***2018 vs. 2021, p = 0.0009. **2016 vs. 2021, p = 0.0010. **2019 vs. 2021, p = 0.0012. **2017 vs. 2021, p = 0.0015. *2022 vs. 2021, p = 0.0117. *2020 vs. 2021, p = 0.0282. 2016–2019 vs. 2020–2023, p = 0.0963 (Fisher’s exact test). [file Image_2.TIFF]
